# Towards Quantum Control with Advanced Quantum Computing: A Perspective

**DOI:** 10.3390/e24121743

**Published:** 2022-11-29

**Authors:** Yongcheng Ding, Yue Ban, Xi Chen

**Affiliations:** 1 International Center of Quantum Artificial Intelligence for Science and Technology (QuArtist) and Department of Physics, Shanghai University, Shanghai 200444, China; 2 Department of Physical Chemistry, University of the Basque Country UPV/EHU, Apartado 644, 48080 Bilbao, Spain; 3 TECNALIA, Basque Research and Technology Alliance (BRTA), 48160 Derio, Spain; 4 EHU Quantum Center, University of the Basque Country UPV/EHU, 48940 Leioa, Spain

**Keywords:** quantum computing, quantum control, quantum simulation, variational quantum circuit, variational quantum algorithm

## Abstract

We propose the combination of digital quantum simulation and variational quantum algorithms as an alternative approach to numerical methods for solving quantum control problems. As a hybrid quantum–classical framework, it provides an efficient simulation of quantum dynamics compared to classical algorithms, exploiting the previous achievements in digital quantum simulation. We analyze the trainability and the performance of such algorithms based on our preliminary works. We show that specific quantum control problems, e.g., finding the switching time for bang-bang control or the digital quantum annealing schedule, can already be studied in the noisy intermediate-scale quantum era. We foresee that these algorithms will contribute even more to quantum control of high precision if the hardware for experimental implementation is developed to the next level.

## 1. Introduction

It is rather hard to tell who contributes most to the eureka moment of quantum computing. Among all researchers who deserve the credit, Richard Feynman pointed out that computing can be a physical process [1]. His landmark paper suggested a universal quantum computer for simulating physics and chemistry. The concept was developed in the following forty years, bringing us to the noisy intermediate-scale quantum (NISQ) era [2,3]. It goes beyond dispute that quantum control plays a vital role in this odyssey by precisely and efficiently manipulating computational units, usually acknowledged as qubits, in the quantum realm. In a nutshell, theorists propose control protocols for constructing universal gate operations on different systems and models, targeting high fidelity, fast operation time, and robustness against noise. Experimentalists implemented optimal protocols for their customized systems and achieved quantum advantages in superconducting circuits [4] and photonic platforms [5].

What about the other way around? In this sense, we formulated the question of whether one can derive quantum control protocols with quantum computing as an alternative approach to classical numerical methods. More specifically, there do exist elegant methods for exact solutions, e.g., invariant-based inverse engineering and counterdiabatic driving in the family of shortcuts to adiabaticity (STA) [6,7,8]. These methods either require a dynamical invariant or a Hamiltonian diagonalization. It does not discourage the theorists but gives rise to variational methods in STA, i.e., proposing Ansätze for the Lagrangian formalism and the engineering on the equations of motion [9]. However, studying numerical methods for quantum control of various systems is still necessary. The reason is that finding appropriate Ansätze is not that trivial, and other quasi-exact protocols do not guarantee a solution of high quality and explicit form for an arbitrary system. Meanwhile, numerical solutions are more than acceptable in experimental implementations, especially when most realistic devices send high-precision digital signals to drive the quantum system. The topic consists of two parts, one as the simulation of quantum dynamics, and the other as the optimization of the control parameters with classical algorithms. Note that here the classical simulation of quantum dynamics is replaced by digital quantum simulation (DQS) [10], which is feasible in universal quantum computers.

With this perspective, we suggest the application of quantum computers as a more efficient numerical method for studying quantum control problems. We explain our framework in detail, review quantum algorithms from the view of quantum control, and outline the further scope of research lines in the rest of this paper.

## 2. Methodology

**Figure 1:**
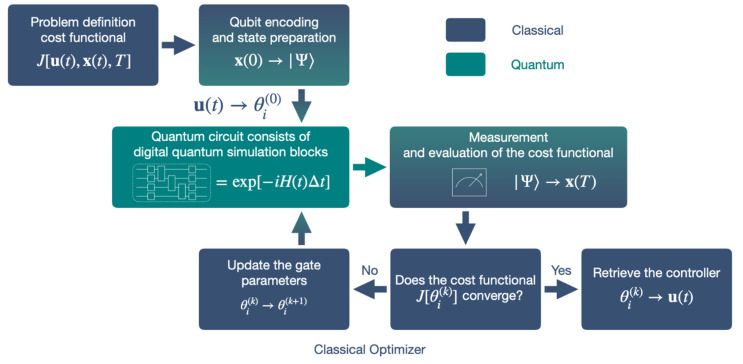
The schematic diagram of using a quantum computer to solve a general quantum control problem numerically. Here, we briefly explain the workflow by starting from the box on the top left. One usually formulates a quantum control problem by minimizing or maximizing a cost functional J[u(t),x(t),T] in a continuous operation time interval t∈[0,T], where u(t) and x(t) are the controller and state, respectively. Our protocol encodes the initial state x(0) into a qubit wave function |Ψ⟩, which is prepared in a quantum computer. Following the Suzuki-Trotter decomposition, we discretize the continuous time into time steps of length Δt, simulating the quantum dynamics with blocks of quantum circuits, which the controller u(t) is also discretized and mapped into gate parameters $\theta_{i}$. After the digital quantum simulation, we measure the qubits to retrieve the classical final state x(T), which allows the evaluation of the cost function. A classical optimizer tunes the gate parameters iteratively until the convergence criteria are satisfied, and outputs the controller u(t) at the end.

The spirit of the proposal is to replace numerical simulation in classical computers with DQS. In this way, we present the head-to-head comparison between classical methods for a better understanding. We show the schematic diagram of the whole workflow in Figure 1, and address the subroutines that differ from classical approaches in the following subsections.

### 2.1. Data Encoding and State Preparation

In the classical simulation of quantum systems, numerical algorithms encode quantum information in different data structures. For example, one usually uses a linked list to store spin configuration in continuous imaginary time for path integral Monte Carlo algorithm to study the quantum phase transition. The simplest data structure for encoding the amplitudes of orthonormal bases in Hilbert space is a complex array, being vectorized for speeding up matrix operations in numerical software by instruction sets. Once the orthogonal bases are chosen, the state initialization in a classical computer is much easier than the state preparation in a quantum computer. The difficulties of initializing the quantum computer from uncoupled ground states of qubits to the target states are different. The initialization circuit depths are usually shallower for spin-1/2 models because of the direct mapping to multiple qubits. On the contrary, the circuit complexity significantly increases if one uses the computational bases as binary codes to store the amplitudes in the lattice space. Regarding the state preparation, we have to prepare the initial wave function in quantum computers for DQS once we confirm the encoding strategy. Arbitrary state preparation is not straightforward in quantum computers. For generality, one can use a variational quantum eigensolver (VQE) [11] to prepare the input state for DQS. The circuit structure for state preparation is problem-designed or based on hardware connection.

### 2.2. Quantum Simulation

The simulation of quantum dynamics becomes an initial value problem that numerically solves a time-dependent Schrödinger equation with given initial conditions. The continuous time is discretized into time steps, which Suzuki–Trotter decomposition gives the approximation evolution [12]. One can evaluate the Trotter error with known time step length and Hamiltonian by the Baker–Campbell–Hausdorff formula. Both classical simulation and DQS evolve the wave function by a time step. Classical simulation, e.g., the splitting operator method, performs matrix multiplications on the vectorized array, while DQS evolves the qubit wave function with quantum gates. Generally, the unitary operation that evolves the qubit wave functions can be represented by a sequence of quantum gates in the universal gate set. Note that it should be distinguished from the universal DQS, or equivalently, the universal quantum computing. Instead of approximating the unitary operation with the Solovay–Kitaev theorem [13], one needs the analytical representation for mapping the systematic parameter to the gate parameters. For example, the evolution of Ising-type ZZ spin interaction for a time step can be implemented by an RZ gate between two CNOTs, in which the rotation angle is half the product of the dimensionless time step and the interaction strength. Thus, the quantum circuit for DQS can be treated as a variational quantum circuit (VQC), in which gate parameters are optimized for calculating the controllers. The application of VQC for quantum simulation is a concerned topic these years [14,15,16,17]. To clarify the novelty of our proposal, we highlight that our protocol finds the unknown solution for quantum control problems with VQCs, instead of simulating desired dynamics with them.

### 2.3. Measurement and Evaluation of the Quantum Function

Another major difference between classical simulation and DQS is that the readout of the result of DQS depends on quantum measurement. Quantum information is destroyed after measurement, requiring repetitive execution of the quantum circuit to estimate the amplitudes. One can reduce the statistical errors by one over the square root of the sampling numbers. The optimization of the VQC aims to minimize a loss function, usually as a fidelity-related cost function in quantum control tasks. The evaluation of the function values is trivial in classical computers (or classical simulators of quantum circuits) by inner producing two vectorized arrays. The evaluation becomes more complicated in quantum computers because of the nature of quantum mechanics. To achieve this goal, one has to design circuits for measurements on certain bases or perform SWAP tests [18] with auxiliary qubits.

### 2.4. Classical Optimization

Like other variational quantum algorithms (VQAs), one needs a classical optimizer to minimize the loss function and return the optimal parameters after convergence to retrieve the controller. One can save quantum resources by using algorithms that do not query too many values of quantum functions to update parameters, e.g., the simultaneous perturbation stochastic algorithm (SPSA) [19], which estimates the gradient with only two values. Note that the dimension of the optimized vector equals the Trotter numbers for single-parameter control and linear growth with the dimension of the controller. Meanwhile, the dimension can be very high for approximating a smooth controller, and its optimization can be simplified by reducing it with Fourier methods or other heuristic strategies [20].

## 3. Examples

### 3.1. Quantum Approximate Optimization Algorithm

The quantum approximate optimization algorithm (QAOA) [21] is regarded as one of the most promising algorithms to achieve quantum advantages in the NISQ era. Inspired by adiabatic quantum computation (AQC) [22], it is a VQA that minimizes the cost function defined by the energy expectation. The QAOA alternatively evolves the problem Hamiltonian and the mixing Hamiltonian on the wave function, driving it from the initial ground state to the final state. The final state encodes the problem’s solution, which is also close to the ground state of the problem Hamiltonian if the gate parameters are optimized appropriately. We highlight that QAOA can also be understood as quantum control of the bang-bang type [23], in which gate parameters are mapped to the evolution time, which is indeed the sum of optimized parameters

$$T=\overset{p}{\underset{i=1}{\sum^{\;}}}\left(\beta_{i}+\gamma_{i}\right)$$

Thus, one can study the corresponding time-optimal bang-bang solution or compare it with other control protocols, e.g., counterdiabatic driving, if the energetic costs are bounded [24]. We point out that QAOA is related to digital adiabatic quantum computing (DAQC) since a QAOA circuit that consists of a large number of p blocks can also be understood as a variational DAQC. To be more specific, one may obtain the schedule of a digital adiabatic quantum computing

$$H\left(t_{i}\right)=A\left(t_{i}\right)\;\underset{i}{\sum }{\hat{\sigma }}_{x}^{i}+B\left(t_{i}\right)H_{p}$$

By

$$A\left(t_{i}\right)=\beta_{i}/\Delta t,B\left(t_{i}\right)=\gamma_{i}/\Delta t$$

where $H_{p}$ is the problem Hamiltonian and Δt=T/p is the length of a Trotter step.

### 3.2. Digital Adiabatic Quantum Computing

The idea of introducing the DAQC paradigm is that it is more flexible to construct arbitrary interactions than quantum annealing, which is also compatible with quantum error correction [25]. In this way, optimizing the parameters in QAOA is equivalent to finding a digitized annealing schedule to minimize the energy excitation, as we explained in the previous subsection. Moreover, if we consider improving the performance of DAQC by introducing counterdiabatic driving, we are indeed working on the proposal that finds an extra control field with a given annealing schedule. In our practice, we introduced STA to DAQC for the GHZ state preparation in a one-dimensional transverse field ferromagnetic Ising model [26]. Its diabatic transitions are suppressed by the first-order NC Ansätz of the ZY + YZ type. We minimized an effective action to obtain the variational coefficients for the extra control field with counterdiabaticity. These STA-enhanced DAQC algorithms are applied to study factorization [27] and portfolio optimization [28]. Although these works were not motivated to obtain a quantum control by quantum computing, subroutines in their workflows have already reproduced the proposal we introduce in this perspective.

### 3.3. Quantum Optimal Control

The quantum harmonic oscillator is the foundation for modeling complex systems and phenomena. Both kinetic and potential terms are quadratic, which means the corresponding unitary operators for evolution are diagonal and of quadratic shape if they are represented in the momentum basis and position basis, respectively. Such operators can be decomposed into a gate sequence with analytical gate parameters [29]. This property satisfies the requirement for the DQS in our proposal. Thus, we studied time-optimal atomic cooling to reveal the connection between the control phase transition and quantum speed limit [30]. Recently, researchers have implemented a similar protocol for controlling the molecular dynamics with VQCs [31], going beyond the quantum harmonic oscillator, realizing Morse potential and rotor models for characterizing hydrogen fluoride and dipole–dipole-coupled carbonyl sulfide, respectively. Numerical experiments also showed that apart from many VQAs, the gradients of the landscape do not vanish with the increasing number of qubits, i.e., there is no barren plateau. In this way, one can still guarantee the trainability of the VQC while reducing the lattice length to improve the simulation accuracy.

## 4. Discussion and Outlooks

Regarding the trainability of VQCs, we analyze the mechanism for maximally convincing the audience that there is no barren plateau. As we can see, the VQC in our framework is highly biased, i.e., the expressibility is very small since it is the Suzuki–Trotter decomposition of a known Hamiltonian. The sharp priors narrow the search space, make the VQC specialized for the task, and result in the absence of a barren plateau [32]. Therefore, increasing the number of qubits for improving the spatial resolution of the DQS or enlarging the system size for DQS does not affect the trainability of the algorithm.

Another satisfying property of the protocol is its robustness against systematic error and quantum noises, which are practically crucial in quantum control. In classical numerical solutions, one usually has to construct a cost function with error sensitivity or other expressions as penalty terms. We point out that the solution obtained from the VQC in a real quantum computer is naturally robust, which mitigates the systematic errors and quantum noises, once the cost function converges under the optimization. The mechanism of such suppression is similar to SPSA for classical optimization, which approximates the gradients, mimicking a certain error at the same time. Its analogy to the classical approach is to introduce errors and noises in the simulation of quantum dynamics, looking for the convergence of the existence of them and the corresponding robust solution [33].

Even though our protocol shows the preceding advantages, one still has to be aware of the stability of Suzuki–Trotter decomposition for DQS, which might affect the validity of the result. For example, the Trotter errors in spin systems with long-range many-body interactions become abnormal [34]. Even minor variations in the Trotter step size lead to sharp changes, which are induced by the structural instability of the Floquet operator. Hence, one should understand the interplay between the properties of the Hamiltonian for DQS and the Trotterized evolution for the specific problem before training the VQC.

After all the examples and analysis, we now go further toward the current feasibility and applications from the theoretical side. Technically, the quantum control of any model with its non-universal DQS proposed can be numerically solved in our framework. Especially, experts from the community of quantum control have enough goals in spin models [35] to be conquered, and numerical simulation on a large scale is not that friendly. Besides the spin models and harmonic oscillators, one can also control the fermionic dynamics, which are responsible for exotic phenomena in quantum chemistry and high energy physics, e.g., the Hubbard model [36] and the SYK model [37], by mapping the fermionic operator into Pauli operators with the Jordan–Wigner [38] transform and the Bravyi–Kitaev transformation [39]. Thus, these techniques enable researchers to investigate quantum control from synthetic chemistry to quantum gravity. We note that a very recent paper solves the preparation for a coherent state [40] by exactly following our protocol, which can be regarded as a timely example.

## 5. Concluding Remarks

We have introduced the hybrid quantum–classical computing framework for numerically solving quantum control problems. The quantum circuit for DQS of a Hamiltonian can be treated as a VQC, if the mappings between the systematic parameters and gate parameters are analytical. By employing the classical optimizers that do not query the quantum function value frequently, one can significantly reduce the shots for quantum measurement before the convergence of the algorithm. Furthermore, the trainability is guaranteed because of the non-existence of barren plateaus in such VQCs. We have listed several applications in the future and the issues that one should be concerned about. We hope this perspective shows the connection between quantum computing and quantum control, which is apart from the widely accepted story of better quantum gates and readouts.
